# Progressively diminished estrogen signaling concordant with increased fibrosis in ectopic endometrium

**DOI:** 10.1093/hropen/hoaf028

**Published:** 2025-05-15

**Authors:** Jichan Nie, Yunhua Yi, Xishi Liu, Sun-Wei Guo

**Affiliations:** Department of Gynecology, Shanghai Obstetrics and Gynecology Hospital, Fudan University, Shanghai, China; Department of Gynecology, Shanghai Obstetrics and Gynecology Hospital, Fudan University, Shanghai, China; Department of Gynecology, Shanghai Obstetrics and Gynecology Hospital, Fudan University, Shanghai, China; Shanghai Key Laboratory of Female Reproductive Endocrine-Related Diseases, Fudan University, Shanghai, China; Shanghai Key Laboratory of Female Reproductive Endocrine-Related Diseases, Fudan University, Shanghai, China; Research Institute, Shanghai Obstetrics and Gynecology Hospital, Fudan University, Shanghai, China

**Keywords:** adenomyosis, endometriosis, ectopic endometrium, estrogen receptor, estrogen signaling, fibrosis

## Abstract

**STUDY QUESTION:**

Do all ectopic endometrial lesions (endometriosis and adenomyosis) universally have activated estrogen signaling?

**SUMMARY ANSWER:**

Estrogen signaling diminishes concordantly with increased fibrosis in ectopic endometrium, with deep endometriotic (DE) lesions exhibiting an estrogen biosynthesis capability and estrogen receptor β (ERβ) expression level comparable to that of control endometrium but having suppressed ERα.

**WHAT IS KNOWN ALREADY:**

Endometriosis and adenomyosis are both estrogen-dependent diseases driven by estrogen-mediated lesional development, progression, and symptom manifestation. Of note, ectopic endometrium is thought to have the ability to synthesize estradiol (E_2_) *in situ* from cholesterol due to upregulation of aromatase (CYP19A1), steroidogenic acute regulatory protein (StAR), 3β-hydroxysteroid dehydrogenase type 2 (HSD3β2), and HSD17β1. In addition to increased estrogen biosynthesis, ERβ and G-protein coupled ER (GPER) are also overexpressed in ectopic endometrium. In particular, the prevailing view holds that prostaglandin E2 plays a vital role in facilitating estrogen biosynthesis and the upregulation of ERβ, positioning itself in the central nexus in a feed-forward loop linking hyperestrogenism and inflammation in all ectopic endometria.

**STUDY DESIGN, SIZE, DURATION:**

After obtaining written informed consent, we collected lesional tissues from 19 patients with ovarian endometriosis (OE) and 20 patients each with adenomyosis (AD) and DE. As controls, normal endometrial tissue samples (CT) were procured from 20 cycling women free of endometriosis and adenomyosis, and age- and menstrual phase-matched with patients in the other groups. Additionally, primary ectopic or control endometrial stromal cells derived from eight subjects in each of the OE, AD, DE, and CT groups were cultured for experiments.

**PARTICIPANTS/MATERIALS, SETTING, METHODS:**

We performed immunohistochemistry and western blotting to assess the expression of proteins key to the estrogen biosynthesis (StAR, HSD3β2, aromatase, and HSD17β1) and estrogen receptors (ERα, ERβ, and GPER). Fibrosis was quantified via Masson trichrome staining. Real-time RT-PCR was performed to assess corresponding gene expression levels. The estrogen concentrations in cell cultures of primary stromal cells derived from different tissues were also measured by ELISA.

**MAIN RESULTS AND THE ROLE OF CHANCE:**

Among all ectopic endometrial tissue samples, the extent of lesional fibrosis was the highest in the DE lesions, followed by the AD and then the OE lesions. The protein and gene expression levels of StAR, HSD3β2, aromatase, and HSD17β1, the four proteins critically involved in estrogen biosynthesis, were significantly higher than in the CT group in OE and AD lesions, but were lowest in DE lesions, which were comparable to that of control endometrium. There was a significantly negative correlation between the expression of these proteins and the extent of lesional fibrosis. Consistently, while the concentration of estrogen in culture supernatants from OE cells was significantly higher than those in CT, it was significantly reduced in AD and DE lesions. In fact, the estrogen concentration in DE cell supernatants was comparable with that in the CT group. The expression of ERβ and GPER was significantly higher in OE and AD lesions than in the CT group and progressively declined with increasing lesional fibrosis; in the DE group, their expression was comparable to the CT group. A significant negative correlation was observed between their expression and the extent of lesional fibrosis. No significant difference in ERα expression was found among different types of ectopic endometrium, but all showed significantly and uniformly lower expression than that of the CT endometrium.

**LIMITATIONS, REASONS FOR CAUTION:**

While diminished estrogen signaling concordant with increased lesional fibrosis was demonstrated, no mechanistic data were provided. In addition, while in this study several genes/proteins known to be key to estrogen signaling were evaluated, some other genes/proteins that are also involved in estrogen signaling, such as other members of the HSD17B family, were not evaluated.

**WIDER IMPLICATIONS OF THE FINDINGS:**

Our findings challenge the prevailing notion of activated *in situ* estrogen signaling in ectopic endometrium of all kinds via the feed-forward loop. As such, there is a need to re-appraise our treatment strategies, especially for lesions that are highly fibrotic and thus well advanced. In addition, our findings can be capitalized to help choose the best treatment modality and to inspire novel therapeutics for endometriosis and adenomyosis.

**STUDY FUNDING/COMPETING INTEREST(S):**

This research was supported in part by grant 82071623 (S.-W.G.) from the National Natural Science Foundation of China and by grant 202440057 (J.N.) from the Clinical Research Project of Shanghai Municipal Health Commission. S.-W.G. is a member of the Scientific Advisory Board of Heranova, BioSciences and of FimmCyte A.G., and has provided consultancy advice to these companies, as well as to Shanghai Huilun Biotechnology, but these activities had no bearing on this work. The other authors have no conflict of interest.

**TRIAL REGISTRATION NUMBER:**

N/A.

WHAT DOES THIS MEAN FOR PATIENTS? Endometriosis and adenomyosis are conditions where abnormal tissue grows outside or within the uterus. These growths have long been thought to thrive on estrogen, a hormone that fuels their growth and worsens symptoms, i.e. pain. It is believed that a molecule called prostaglandin E_2_ (PGE_2_) plays a key role in creating a harmful cycle where estrogen and inflammation feed off each other in all types of these growths.

However, we previously found that the action of PGE_2_ is diminished when these growths are heavily scarred (i.e. fibrotic). Indeed, our current research shows that not all growths behave the same way. The role of estrogen depends heavily on how much scarring (i.e. fibrosis) the tissue has.

Endometrial growths in the ovaries (ovarian endometrioma) or uterine muscle (adenomyosis) do have high estrogen activity, which likely drives the symptoms. However, deep endometriosis (growths in areas like the bowel or bladder) shows very low estrogen activity, similar to that in healthy uterine tissue. This seems to be because these deep growths are often heavily scarred which diminishes the actions of PGE_2_

Current hormonal therapies (e.g. birth control, GnRH agonists) aim to block estrogen. But if scarred, deep growths do not use estrogen to grow, then these drugs may not work well for them. This helps explain why some patients do not improve with standard treatments.

In short, this study highlights the need for personalized treatment plans, depending on how much scarring there is in these abnormal tissues, and opens doors to better, more effective options for patients.

## Introduction

Endometriosis and adenomyosis are among the most prevalent gynecological disorders in women of reproductive age, second only to uterine fibroids (Zimmermann *et al.*, 2012). Reported prevalence ranges from 6% to 10% (Giudice and Kao, 2004) for endometriosis to 20–34% for adenomyosis (Naftalin *et al.*, 2012; Pinzauti *et al.*, 2015; Chapron *et al.*, 2020; Kho *et al.*, 2021; Vannuccini *et al.*, 2024). Both diseases are characterized by the presence of ectopic endometrium and share overlapping symptomology, including dysmenorrhea, pelvic pain, infertility and, in adenomyosis in particular, heavy menstrual bleeding. These symptoms contribute to a significant reduction in work productivity and quality of life, and an increased healthcare burden on patients globally (Nnoaham *et al.*, 2011; Simoens *et al.*, 2011, 2012).

While the pathogenesis and pathophysiology of these two diseases are still shrouded largely in mystery, estrogen dependence is a well-established hallmark. Estrogen drives lesional development, progression, and symptom manifestation (Giudice and Kao, 2004; Kitawaki, 2006). Unlike normal endometrium, ectopic endometrium has the ability to synthesize estradiol (E_2_) *in situ* from cholesterol thanks to upregulation of two of the most important enzymes involved in the process of estrogen biosynthesis, aromatase (CYP19A1), and steroidogenic acute regulatory protein (StAR) (Bulun *et al.*, 2005). Additionally, 3β-hydroxysteroid dehydrogenase type 2 (HSD3β2), which converts dehydroepiandrosterone to androstenedione, that can be further converted to estrone (E_1_) by aromatase, and HSD17β1, which converts E_1_ into E_2_, a more potent estrogen, are also aberrantly upregulated (Attar *et al.*, 2009; Smuc *et al.*, 2009). In addition to increased estrogen biosynthesis, ectopic endometrium also exhibits overexpression of estrogen receptors, particularly ERβ and G-protein coupled ER (GPER, also called GPR30) (Brandenberger *et al.*, 1999; Samartzis *et al.*, 2012). Prostaglandin E_2_ (PGE_2_) plays a vital role in facilitating estrogen biosynthesis and the upregulation of ERβ (Bulun *et al.*, 2005; Attar *et al.*, 2009), positioning itself in the central nexus in a feed-forward loop linking hyperestrogenism and inflammation (Bulun *et al.*, 2005).

Consequently, the mainstay treatment of today for both diseases is, aside from surgery, hormonal medications, which are aimed at altering the menstrual cycle in order to produce a pseudo-pregnancy, pseudo-menopause, or chronic anovulation in an acyclic, hypo-estrogenic environment (Barbieri, 1992; Brosens, 1997). Postoperative hormonal regimens are also standard to mitigate the risk of recurrence (Guo, 2009).

The hormonal drugs are effective in managing both diseases, but these drugs have various side effects (Vercellini *et al.*, 2018b), so much so that a strong undercurrent dismissive of hormonal drugs has surfaced recently (Burla *et al.*, 2021). Unfortunately, the development of non-hormonal therapeutics for either endometriosis or adenomyosis so far has not been successful (Guo, 2014, 2023).

Despite the vital roles of estrogen signaling in ectopic endometrium, clinical trials targeting estrogen signaling have yielded puzzling outcomes. A phase II clinical trial launched in 1999, with great fanfare, on the use of fulvestrant (a potent pure antiestrogen) for endometriosis, ended unceremoniously without a trace (Guo and Olive, 2007). Similarly, a 2005 trial of raloxifene (an estrogen antagonist) was terminated early and unexpectedly due to accelerated pain recurrence and surgical reintervention in treated patients (Stratton *et al.*, 2008). More inexplicably, despite the recognized centrality of PGE_2_ signaling in the development of endometriotic lesions (Wu *et al.*, 2010; Lai *et al.*, 2019), and perhaps in adenomyotic lesions as well, surprisingly there are few reports on the efficacy of COX-2 inhibitors (Brown *et al.*, 2017). Equally inexplicably, adenomyosis (AD) and certain subtypes of endometriosis, such as deep endometriosis (DE), do not respond to hormonal treatment as well as ovarian endometrioma (OE) (Stratopoulou *et al.*, 2021; Vannuccini *et al.*, 2022), and aromatase inhibitors show no superior efficacy (Csirzo *et al.*, 2024).

These seemingly incongruent, and sometimes inexplicable, findings call for a more careful scrutiny of the estrogen signaling in ectopic endometrium. This is particularly necessary and timely in light of recent reports that the PGE_2_ signaling is diminished as the extent of lesional fibrosis increases (Huang *et al.*, 2022b,c; Yi *et al.*, 2025). Traditionally viewed as the elephant in the room, lesional fibrosis is now increasing recognized as an important feature of endometriosis (Guo, 2018; Vigano *et al.*, 2018) and adenomyosis (Guo, 2022). This is particularly relevant since the extent of fibrosis in ectopic endometrium is associated with the lesional developmental stage (Guo *et al.*, 2015; Zhang *et al.*, 2016) and severity of dysmenorrhea (Liu *et al.*, 2018a; Nie *et al.*, 2022), and is implicated to be responsible for heavy menstrual bleeding in adenomyosis (Huang *et al.*, 2022a), yet refractory to medical treatment (Liu *et al.*, 2018b; Shen *et al.*, 2025).

In this study, we aimed to evaluate expression levels of genes/proteins critically involved in estrogen biosynthesis (StAR, aromatase, HSD3β2, and HSD17β1) and receptors (ERα, ERβ, and GPER) in OE, AD, and DE lesions relative to control endometrium. While these disease entities/subtypes may well have different pathogenesis and pathophysiology, they also exhibit different degrees of fibrosis (Liu *et al.*, 2018b). Therefore, juxtaposing these different types of lesions may provide us with more insight into the estrogen signaling than investigating one particular type alone. We hypothesized that the estrogen signaling diminishes concordant with the increasing lesional fibrosis. This study was undertaken to test this hypothesis.

## Materials and methods

### Ethics statement

This research was approved by the Institutional Review Board of Shanghai OB/GYN Hospital, Fudan University (No. 2020-85). All patients participating in this study signed a written informed consent form, agreeing to the collection of specimens and medical history data. Furthermore, the design, data collection, and analysis of this study adhered to the Helsinki Declaration, the Committee on Publication Ethics (COPE) guidelines (http://publicationethics.org/), and the RECORD statement.

### Patients and specimens

Leveraging our prior finding on diminished PGE_2_ signaling (Yi *et al.*, 2025), we utilized the same patient cohort and their tissue samples for immunohistochemistry (IHC) analysis. Briefly, after informed consent, we collected clinical data, by a combination of history taking, gynecological examination, ultrasound, and MRI, from 59 patients diagnosed with endometriosis or adenomyosis and indicated for surgery at our hospital between September 2020 and April 2021; all patients received surgical and pathological confirmation of their diagnosis. Among these, 19 cases were classified as OE, while 20 each were diagnosed with deep endometriosis (DE) and diffuse adenomyosis (AD). Diffuse AD was selected to minimize heterogeneity and facilitate sample collection. Given that we had a moderate sample size, it made more sense to focus on one particular type to eliminate possible heterogeneity between different types of AD.

During surgery, lesional tissues, such as the cyst wall tissues of OE, of approximately 0.5–1.0 cm in diameter were collected for subsequent experiments (Nie *et al.*, 2018; Qiu *et al.*, 2023). All collected specimens were evaluated by experienced pathologists for histological confirmation. Additionally, for controls, we recruited 20 patients who underwent total hysterectomy due to high-grade squamous intraepithelial lesions of the cervix (HSIL), with no other benign or malignant gynecological diseases, and after informed consent, collected their full-thickness endometrial tissue samples, including both the basal and functional layers. The four groups were age- and menstrual phase-matched (in frequency). All recruited subjects were premenopausal and had not received hormonal medication for at least 3 months prior to surgery. For all recruited participants, the severity of dysmenorrhea was assessed using a 10-mm visual analog scale (VAS), where 0 indicated no pain and 10 indicated intolerable pain for subsequent analyses. The collected tissue samples, along with clinical data, were used for immunohistochemical staining and Masson staining and part of results on PGE_2_ signaling has been reported previously (Yi *et al.*, 2025).

In addition, using (the previously reported) eight lesion samples each from patients with OE, AD, and DE and eight control (CT) endometrial tissue samples from women with HSIL but free of other gynecological diseases who underwent hysterectomy (Yi *et al.*, 2025), we quantitated the mRNA abundance of COL1A1 (encoding collagen I), as well as STAR (encoding StAR), HSD3B2 (encoding HSD3β2), CYP19A1 (encoding aromatase), HSD17B1 (encoding HSD17β1), ESR1 (encoding ERα), ESR2 (encoding ERβ), and GPER, by RT-PCR in these samples.

Moreover, for quantification of protein expression levels of StAR, HSD3β2, aromatase, HSD17β1, ERα, ERβ, and GPER, we procured, after obtaining informed consent, additional endometriotic/adenomyotic lesion samples from eight each OE, AD, and DE premenopausal patients as well as control samples from eight CT (HSIL) patients who underwent hysterectomy but free of other uterine abnormalities. The patients in these four groups were matched for both menstrual phase and age.

All recruited patients were non-smokers, and did not receive any infertility treatment or any hormonal or anti-diabetic medication 3 months prior to the surgery. Furthermore, there was no family or previous history of deep vein thrombosis, blood clotting issues, or a genetic predisposition to malignancies. A flow chart illustrating the study design, including the inclusion and exclusion criteria, is shown in Supplementary Fig. S1.

### IHC and Masson trichrome staining

The tissue samples were fixed in formalin and then embedded in paraffin. Each collected tissue block was consecutively cut into 4 µm sections. The first section was stained with hematoxylin and eosin for pathological confirmation, and the remaining sections were used for IHC analyses.

Routine deparaffinization and phosphate-buffered saline (PBS) washing steps were performed. For antigen retrieval, the slides were placed in citrate buffer (pH 6.0) and heated at 98°C for 30 min, then allowed to cool to room temperature. Then, 5% normal goat serum was added for antigen blocking. Primary antibodies against StAR, HSD17β1, aromatase, HSD3β2, ERα, ERβ, and GPER were incubated on the sections overnight at 4°C. Information regarding these antibodies is provided in Table 1.

**Table 1.** Information on the antibodies used in the immunohistochemistry analyses and western blotting.

Antibody name	Vendor name and location	Catalog number	Concentration (IHC/WB)
StAR	ProteinTech Group (Chicago, IL, USA)	12225-1-AP	1:200/1:1000
HSD3β2	Abcam (Cambridge, UK)	ab154385	1:400/1:2000
Aromatase	Abcam (Cambridge, UK)	ab18995	1:200/1:1000
HSD17β1	ProteinTech Group	25334-1-AP	1:1000/1:3000
Erα	Abcam	ab108398	1:200/1:2000
Erβ	Abcam	ab288	1:200/1:2000
G-protein coupled ER	Bioss (Woburn, MA, USA)	Bs-1380R	1:200/1:2000

IHC, immunohistochemistry; WB, western blotting.

After slides were thoroughly rinsed, they were incubated for 30 min at room temperature with horseradish peroxidase (HRP)-conjugated secondary antibodies against rabbit/mouse antibodies (Shanghai Sun BioTech Company, Shanghai, China). The bound antibody complexes were counterstained with hematoxylin (30 s) after being stained with diaminobenzidine for around 1–2 min, or until they were ready for microscopic inspection.

To ensure consistency, accuracy, and reliability, three to five randomly selected images of high-power field from each stained section were captured with a microscope ( × 400 amplification; Olympus BX53, Olympus, Tokyo, Japan) to obtain a mean value for each IHC measure/protein counted by Image-Pro Plus 6.0 (Media Cybernetics, Bethesda, MD, USA). The positive staining was evaluated using a semi-quantitative scoring system. Briefly, IHC parameters assessed in the selected areas of endometrium included (i) integrated optical density (IOD); (ii) total stained area (*S*); and (iii) mean optical density (MOD), which is defined as MOD = IOD/*S*, equivalent to staining levels of all positive cells. The resulting number of data points was stated when appropriate. All histological, histochemical, and IHC scorings were performed by a single observer (Y.Y.). To maintain objectivity, the observer was completely blinded to the group identity of all slides that she was evaluating.

As previously reported (Shen *et al.*, 2009; Ding *et al.*, 2015; Long *et al.*, 2016), the average optional density values were then computed using Image Pro-Plus 6.0 software (Media Cybernetics, Inc., Bethesda, MD, USA). Since both the epithelial and the stromal components of ectopic endometrium were stained positive for StAR, HSD3β2, aromatase, HSD17β1, ERα, ERβ, and GPER, the IHC scoring was done separately for the two components and reported separately.

For negative controls, we utilized OE lesion tissues and used immunoglobulin G from rabbit serum (Sigma, Darmstadt, Germany) in lieu of the primary antibody using the same concentration as that of the primary antibody for staining and followed all other steps in complete accordance with the IHC procedures. StAR, HSD17β1, aromatase, HSD3β2, ERα, ERβ, and GPER are reported to be expressed in OE lesions (Kitawaki *et al.*, 1997; Tian *et al.*, 2009; Sroyraya *et al.*, 2018). Therefore, their immunostaining in OE lesions served as positive controls. Representative photomicrographs of negative and positive staining controls are provided in Supplementary Fig. S2.

Masson trichrome staining was used to detect collagen fibers in tissue samples and to quantify the extent of lesional fibrosis as previously reported (Yi *et al.*, 2025). Tissue sections were deparaffinized in xylene and rehydrated in a graded alcohol series and then were immersed in Bouin solution at 37°C for 2 h. The Bouin solution was made with 75 ml of saturated picric acid, 25 ml of 10% formalin (w/v) solution, and 5 ml of acetic acid. The tissue sections were stained using Masson’s trichrome staining kit (Baso, Wuhan, China) following the manufacturer’s instructions. Slides were then mounted and evaluated under an Olympus microscope (Olympus) at X200 magnification, capturing four to five different fields of sections. The areas of the collagen fiber layer stained in blue in proportion to the entire field of the ectopic implants were calculated using Image Pro-Plus 6.0 (Media Cybernetics, Inc). Masson staining parameters assessed in the detected area included: (i) IOD, (ii) total stained area (*S*), and (iii) MOD, which was defined as MOD = IOD/*S* and used as the extent of lesional fibrosis.

### Cell culture

While IHC analysis can be informative regarding which cell types and where within the cells are positively stained and the staining intensity, it only quantitates the staining intensity semi-quantitatively. In order to see at which level (transcriptional vs translational) the expression becomes aberrant, quantification of gene and protein expression levels using primary cells is needed. Since traditionally the investigation of estrogen signaling in ectopic endometrium has been nearly exclusively focused on the stromal component (Noble *et al.*, 1996; Tsai *et al.*, 2001), we used primary stromal cells derived from eutopic and ectopic endometrial tissues.

We used the standard procedures (Zhan *et al.*, 2020; Park *et al.*, 2023) with minor modifications to extract and culture the endometriotic/endometrial stromal cells. Briefly, endometriotic and endometrial tissues were cut into small pieces less than ∼1 mm^3^ in size, and submerged in Dulbecco’s modified Eagle’s medium (DMEM)/F12 (HyClone Laboratories, Logan, UT, USA) containing type IV collagenase (2.5 mg/ml; Gibco Laboratories, Grand Island, NY, USA), and gently shaken for an hour at 37°C to facilitate enzymolysis. The tissues were then separated using serial filtration through two successive gauge sieves, measuring 100 and 40 μm, respectively. The sedimentation containing stromal cells was suspended in DMEM/F12 and plated onto 6-cm dishes after the filtrated cells were centrifuged. Other suspending components were washed away with PBS following a 24-h incubation period at 37°C to allow for stromal cell adhesion. DMEM/F12 supplemented with 10% fetal bovine serum (Hyclone Laboratories) was used to cultivate the cells. Following the proliferation to 80–90% confluence, the cells were separated using 0.25% trypsin, replated at 5 × 10^5^ cells/well onto fresh dishes, and incubated at 37°C in a humidified environment with 5% CO_2_ in the air. The primary stromal cells were verified through positive immunohistochemical staining of vimentin and negative staining of cytokeratin 7, von Willebrand factor (vWF), Desmin, and follicle-stimulating hormone receptor (FSHR), ensuring that the purity exceeded 95% (Supplementary Fig. S3) (Chiappini *et al.*, 2022). Primary cells within 3–10 culture passages were used.

### Real-time RT-PCR

Following PBS (HyClone Laboratories) washing, the EZ-press Cell to cDNA Kit (EZ Bioscience, Roseville, MN, USA) was used to lyse the primary stromal cells and convert mRNA into cDNA in accordance with the manufacturer’s instructions and thermal profile. The abundance of mRNA was then assessed using a Real-Time PCR instrument (Life Technologies, Carlsbad, CA, USA) and the 2 ×  Color SYBR Green qPCR Master Mix Kit (EZ Bioscience, Roseville, MN, USA). The mRNA abundance of COL1A1, STAR, HSD17B1, CYP19A1, HSD3B2, ESR1, ESR2, and GPER were evaluated. For normalization, the geometric mean of the GAPDH readings was employed. The fold change in relation to the control (CT) group was used. Supplementary Table S1 contains the list of the genes and primers used in this study.

### Western blot analysis

The primary cells were extracted from T25 cell culture flasks after a few days of culture by exposing them to 5% trypsin/EDTA (Gibco Laboratories, Carlsbad, CA, USA) for 2 min at 37°C. Cells were scraped and their total proteins were extracted in a radio-immunoprecipitation assay buffer (Thermo Fisher Scientific, Pittsburgh, PA, USA) following washing with PBS (HyClone Laboratories). The bicinchoninic acid protein quantitative analysis kit (P0010S; Beyotime, Shanghai, China) was then used to measure the protein concentration in each sample. Following loading onto pre-cast 10% SDS–PAGE gels (PG112; Epizyme, Shanghai, China) and subsequent electrophoresis, the protein samples were transferred to PVDF membranes (Bio-Rad, Hercules, CA, USA). After incubating the membranes with the primary antibodies specified in Table 1 for an entire night at 4°C, they were treated for an hour at room temperature with HRP-labeled secondary antibodies (Arigo, Hsinchu, Taiwan, China). The images were then digitalized on the Image Quant LAS 4000 mini (GE Healthcare, Marburg, MA, USA) and developed using an enhanced chemiluminescence reagent (NCM Biotech; Suzhou, Jiangsu, China). Image J software was employed to quantify the images. GAPDH was used for loading control. All results were presented as fold changes in protein expression relative to the control group.

### ELISA

Cells were seeded to culture plates at a density of 1 × 10^5^ viable cells per well and incubated in 150 μl of DMEM/F-12 at 37°C in a humidified atmosphere of 95% air and 5% CO_2_ for 24 h to allow cell attachment to the culture plates. The culture medium was then discarded, and the cells were washed twice with PBS. All culture plates were further incubated at 37°C in 5% CO_2_ for an additional 48 h, after which the culture medium was collected and stored at −80°C for subsequent ELISA analysis (Lu *et al.*, 2012).

Following the manufacturer’s instructions, the Human 17β-estradiol ELISA Kit (Labor Diagnostika Nord GmbH, Nordhorn, Germany) was used to measure the concentration of E_2_ in the supernatants. The detection range of the assay is between 10.6 and −2000 pg/ml. Each well was filled with the aqueous samples. An antibody–antigen–enzyme–antibody complex was created when aqueous E_2_ combines with an antibody tagged with HRP. The HRP-enzyme-catalyzed tetramethylbenzidine (TMB) substrate turned blue when the TMB substrate solution was added after thorough washing with the prepared washing solution provided in the kit. A solution of sulfuric acid was added to stop the reaction, and the color shift was detected using spectrophotometry at 450 nm. A standard curve was created by drawing a curve between the places where the optical densities (ODs) of the standards and the standard concentration were displayed on a graph (*R*^2^ = 0.99). The OD of the samples was then compared to the standard curve to determine the E_2_ concentration. Three measurements of the E_2_ concentration were made for each sample, and the average was used for the E_2_ concentration in that sample.

### Statistical analysis

Wilcoxon’s rank test was employed to compare two independent groups. Pearson’s or Spearman’s rank correlation coefficient was used when evaluating correlations between two variables when both variables were continuous or when at least one variable was ordinal. To evaluate which factors were associated with the staining levels, multiple linear regression analysis was used, incorporating age, menstrual phase, co-occurrence of adenomyosis or of uterine fibroids, and whether the patient had OE, AD, or DE as co-variables. Since we incorporated about seven to eight covariables and we had a total sample size of about 80, this is still within the rule of thumb, i.e. each covariable would require about sample size of 10. To fully use the data, linear regression was used for analyzing *in vitro* gene and protein expression data. *P*-values of <0.05 were considered statistically significant. All computations were made with R version 4.4.2 (R Core Team, 2021).

## Results

### Differential extent of lesional fibrosis in different types of ectopic endometrium

To gain further insight into the PGE_2_−estrogen feed-forward loop, we used the same set of tissue samples that we used for reporting the PGE_2_ signaling in different types of ectopic endometrium (Yi *et al.*, 2025). For completeness, the characteristics of recruited patients with OE, AD, and DE, as well as those controls, are listed in Table 2. The three groups of patients were comparable to controls with respect to age and menstrual phase (Table 2). However, DE patients had significantly lower parity as compared with controls. In addition, all three groups had significantly more severe dysmenorrhea than the control group (Table 2). The VAS scores on dysmenorrhea were highly correlated with the verbal rating scale scores (Spearman’s *r* = 0.99, *P* < 2.2 × 10^−16^). A multiple linear regression on the VAS scores using age, parity, group identity (OE, AD, or DE), whether there is co-occurrence of OE, or AD, or just AD, or co-occurrence of uterine fibroids identified that being OE, AD, and DE, and with co-occurrence with DE (for OE or AD patients), were all positively associated with higher VAS scores (*R*^2^ = 0.52, all four *P*-values ≤ 0.035).

**Table 2.** Characteristics of recruited patients with ovarian endometriomas (OE), adenomyosis (AD), deep endometriosis (DE), and the control group (CT).

Variable	CT	OE	AD	DE	Statistical significance
	(n = 20)	(n = 19)	(n = 20)	(n = 20)	
**Age (in years)**					
Mean±SD	41.7 ± 4.3	40.2 ± 5.1^NS^	40.9 ± 5.0^NS^	40.7 ± 6.7^NS^	0.60
Median	41.5	41.0	41.0	41.0	
[Min, Max]	[35.0, 52.0]	[30.0, 50.0]	[33.0, 9.0]	[30.0, 49.0]	
**Menstrual phase**					
Proliferative	10 (50.0%)	7 (36.8%)^NS^	10(50.0%)^NS^	10 (50.0%)^NS^	0.79
Secretory	10 (50.0%)	12 (63.2%)	10 (50.0%)	10 (50.0%)	
**Parity**					
0	0 (0.0%)	2 (10.5%)^NS^	4 (20.0%)^NS^	6 (30.0%)**	0.12
1	16 (80.0%)	13 (68.4%)	14 (70.0%)	13 (65.0%)	
≥2	4 (20.0%)	4 (21.1%)	2 (10.0%)	1 (5.0%)	
**Severity of dysmenorrhea**					
None	20 (100%)	6 (31.6%)***	4 (20.0%)***	3 (13.0%)***	
Mild	0 (0%)	7 (36.8%)	6 (30.0%)	4 (20.0%)	1.9×10^−8^
Moderate	0 (0%)	6 (31.6%)	7 (35.0%)	6 (30.0%)	
Severe	0 (0%)	0 (0%)	3 (15.0%)	7 (35.0%)	
**VAS score on dysmenorrhea**					
Mean±SD	0.0 ± 4.3	2.7 ± 2.2***	4.1 ± 2.8***	5.0 ± 2.9***	
Median	0	3	3.5	5.5	2.0×10^−7^
[Min, Max]	[0, 0]	[0, 6]	[0, 9]	[0, 9]	
**rASRM stage**					
I	N.A.	0 (0.0%)	N.A.	2 (10.0%)	
II		0 (0.0%)		0 (0.0%)	N.A.
III		8 (42.1%)		10 (50.0%)	
IV		11 (57.9%)		8 (40.0%)	
**Co-occurrence of ovarian endometrioma**					
Yes	0 (0%)	19 (100%)	4 (20.0%)	10 (50.0%)	3.1×10^−12^
No	20 (100%)	0 (0%)	16 (80.0%)	10 (50.0%)	
**Co-occurrence of adenomyosis**					
Yes	0 (0%)	3 (15.8%)	20 (100%)	7 (35.0%)	7.7×10^−13^
No	20 (100%)	16 (84.2%)	0 (0%)	13 (65.0%)	
**Co-occurrence of deep endometriosis**					
Yes	0 (0%)	0 (0%)	1 (5.0%)	20 (100%)	<2.2×10^−16^
No	20 (100%)	19 (100%)	19 (95.0%)	0 (0%)	
**Co-occurrence of uterine fibroids**					
Yes	0 (0%)	0 (0%)^NS^	10(50.0%)***	8 (40.0%)**	4.2×10^−6^
No	20 (100%)	19 (100%)	10 (50.0%)	12 (60.0%)	

Most content of this table has been reported previously (Yi *et al.*, 2025), but here the data on VAS scores on dysmenorrhea are presented and the entire table is presented for completeness.

rASRM, Revised American Society for Reproductive Medicine endometriosis classification system; VAS, visual analog scale.

* *P* < 0.05;

** *P* < 0.01;

*** *P* < 0.001; NS: *P* > 0.05.

As previously reported, the extent of fibrosis in all three types of ectopic endometrium was significantly elevated as compared with the extent of endometrial fibrosis in CT samples, so were the lesional staining levels of α-SMA, with the DE lesions exhibiting the highest staining levels, followed by AD and OE lesions (Yi *et al.*, 2025). These results were confirmed by RT-PCR finding that the gene expression levels for *COL1A1* (encoding collagen I) and *ACTA2* (encoding α-SMA) in primary endometriotic stromal cells derived from OE, AD, and DE tissue samples were all significantly higher than that of endometrial stromal cells from controls, with the highest in DE lesions, followed by AD and then OE lesions (Yi *et al.*, 2025).

**Figure 1.**
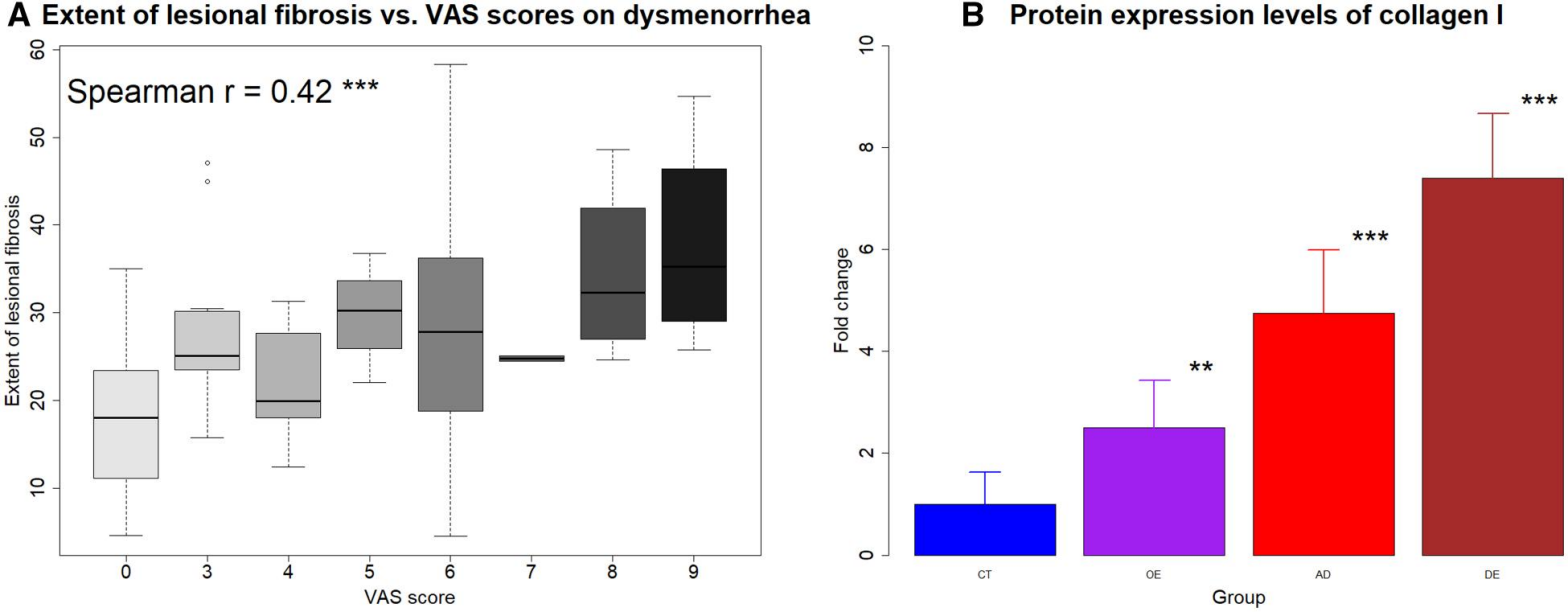
**Relationship between lesional fibrosis and dysmenorrhea severity, and protein expression levels of collagen I**. Boxplot showing the relationship between the extent of lesional fibrosis and the VAS scores on dysmenorrhea (**A**). In (**B**), the protein expression levels of collagen I in the four different groups are presented. CT, control endometrium; OE, ovarian endometriomas; AD, adenomyosis; DE, deep endometriosis; VAS, visual analog scale. Symbols for statistical significance levels: **P < 0.01; ***P < 0.001.

Within patients with ectopic endometrium, the extent of lesional fibrosis correlated positively with the severity of dysmenorrhea (Spearman’s *r* = 0.42, *P* = 0.0008; Fig. 1A), consistent with previous reports (Nie *et al.*, 2022; Yi *et al.*, 2025).

To further validate our results, we next evaluated, by western blotting, the protein expression levels of collagen I from primary endometriotic stromal cells derived from different lesion tissues as well as from normal endometrium using a different set of tissue samples. Consistent with the Masson staining and the PCR results, the protein expression level of collagen I was highest in DE lesions, followed by AD and then OE lesions, with all groups showing significantly higher levels than CT tissues (all *P*-values ≤ 0.0006; Fig. 1B).

### Diminished expression of estrogen biosynthesis enzymes concomitant with increasing extent of lesional fibrosis

**Figure 2.**
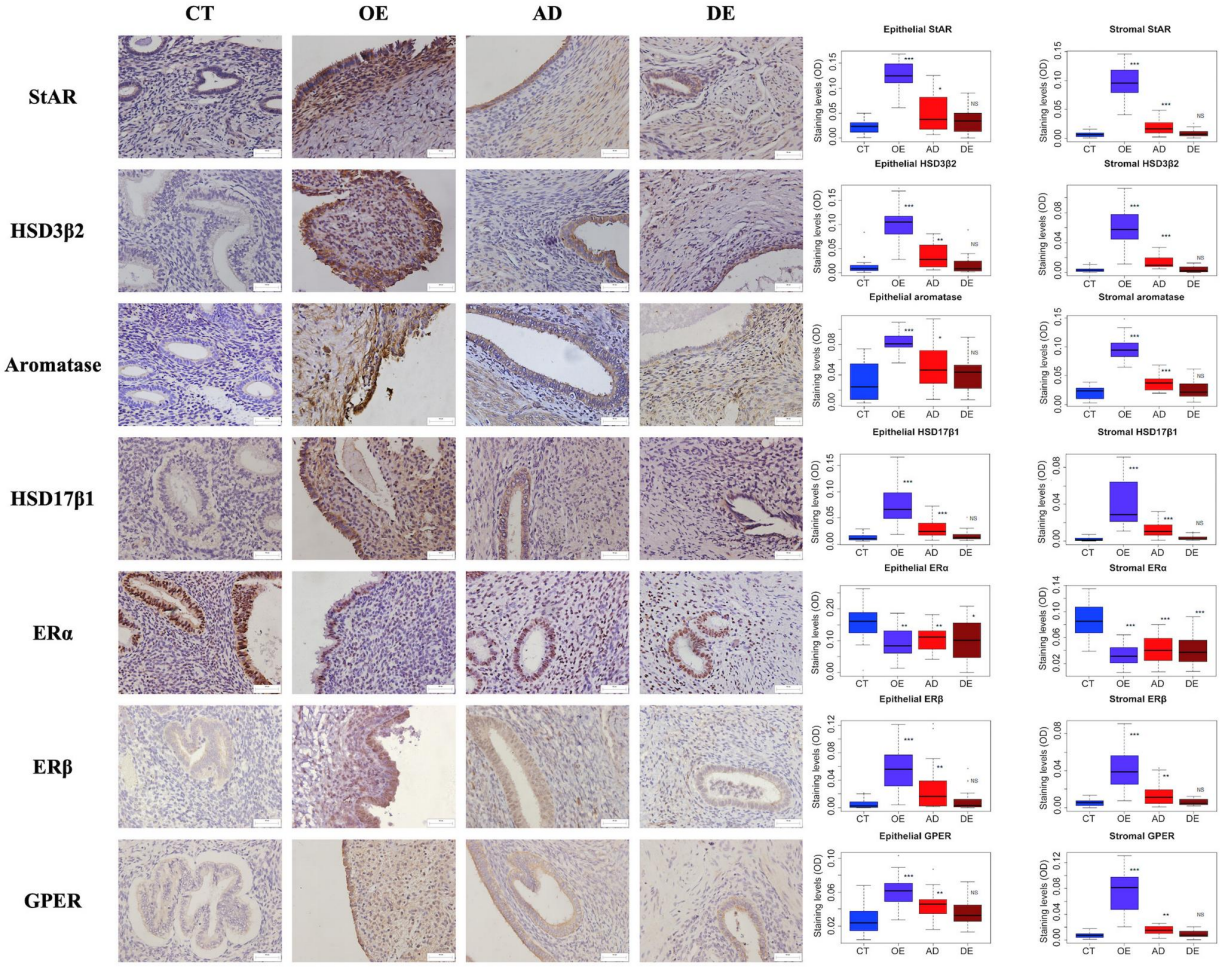
**Results of immunohistochemistry analysis of proteins involved in the estrogen signaling pathway**. The representative photomicrographs (left panel) of the immunostaining of StAR, HSD3β2, aromatase, HSD17β1, and estrogen receptors ERα, ERβ, and G-protein coupled ER (GPER) in normal endometrium (CT), ovarian endometrioma (OE), adenomyosis (AD), and deep endometriosis (DE) tissue samples, along with data summary (right panel). Sample sizes: CT: n = 20; OE: n = 19; AD: n = 20; DE: n = 20. Magnification: × 400. Scale bar= 50 μm. Symbols for statistical significance levels: NS: *P* > 0.05; *P < 0.05; **P < 0.01; ***P < 0.001 (by Wilcoxon’s rank test, *: compared with the CT group).

We next evaluated the immunoreactivity against proteins involved critically in estrogen biosynthesis and reported to be overexpressed in ectopic endometrium, such as StAR, HSD3β2, aromatase, and HSD17β1. In addition, we also evaluated the immunoreactivity against three estrogen receptors, ERα, ERβ, and GPER. The positively immunostained cell types and locations are presented in Table 3. For all four proteins involved in estrogen production, both OE and AD lesions had significantly higher staining levels than that of CT tissues in both epithelial and stromal components (all *P*-values ≤ 0.023; Fig. 2), but DE lesions had comparable staining to CT tissues (all four *P*-values ≥ 0.091). Multiple linear regression incorporating age, menstrual phase, parity, and the co-occurrence with OE, AD, DE, and UF as co-variables confirmed these results (Table 3). Of note, the DE lesions had comparable immunoreactivity against StAR, HSD3β2, aromatase, and HSD17β1 to the CT samples (Table 3).

**Table 3.** List of immunohistochemistry markers, along with their distribution in different cell types, and the location in which the scoring was performed, in conjunction with the results of multiple linear regression analyses.

Marker name	Cellular component(s)	Location of the staining	OE	AD	DE	R^2^	Remark
Extent of lesional fibrosis	Entire lesion	Entire lesion	↑***	↑***	↑***	0.69	Co-occurrence of UF↑*
StAR	Epithelium&stromal	C	↑***	↑**	NS	0.66	
			↑***	↑*	NS	0.84	
HSD3β2	Epithelium&stromal	C	↑***	↑**	NS	0.67	
			↑***	↑*	NS	0.72	
Aromatase	Epithelium&stromal	M	↑***	↑*	NS	0.42	Secretory phase↑*
			↑***	↑**	NS	0.78	Age ↓*
HSD17β1	Epithelium&stromal	C	↑***	↑***	NS	0.65	Co-occurrence with OE ↑**
			↑***	↑***	NS	0.70	
ERα	Epithelium&stromal	N + C+M	↓**	↓**	↓**	0.16	
			↓***	↓***	↓***	0.48	
ERβ	Epithelium&stromal	N	↑***	↑***	NS	0.50	Secretory phase↓**
			↑***	↑**	NS	0.61	
G-protein coupled ER	Epithelium&stromal	M	↑***	↑***	NS	0.36	
			↑***	NS	NS	0.73	

Results from the epithelial and the stromal components were analyzed separately. The results on the epithelial component are shown in the first row of each cell (except for tissue fibrosis, which was scored for the entire lesion area), while those on the stromal component are shown in the second row.

C, cytoplasm; N, nuclear; M, membrane; AD: adenomyosis; DE, deep endometriosis; OE, ovarian endometriomas; UF, uterine fibroids.↓: significant decrease; ↑: significant increase. The number of *s indicates the significance level, e.g.

* *P* < 0.05;

** *P* < 0.01;

*** *P* < 0.001.

Similar to the above four proteins, both OE and AD lesions had significantly higher ERβ and GPER staining levels in both epithelial and stromal components than that in CT tissues (all *P*-values ≤0.0051; Fig. 2), but DE lesions had comparable staining to CT tissues (all four *P*-values ≥ 0.12). Multiple linear regression yielded very similar results, except GPER staining in the stromal component in AD lesions was also comparable to that of CT tissues (Table 3). In contrast, all three types of ectopic endometrium displayed significantly lower ERα staining levels in both epithelial and stromal components than that in CT tissues (all *P*-values ≤ 0.014; Fig. 2), which was also confirmed by multiple linear regression analysis (Table 3). Of note, with the only exception of ERα (*r* = 0.16, *P* = 0.16), the staining levels of the other six proteins were positively correlated between the epithelial and the stromal component (all *r* ≥ 0.54, all *P*-values ≤ 3.6 × 10^−7^).

**Figure 3.**
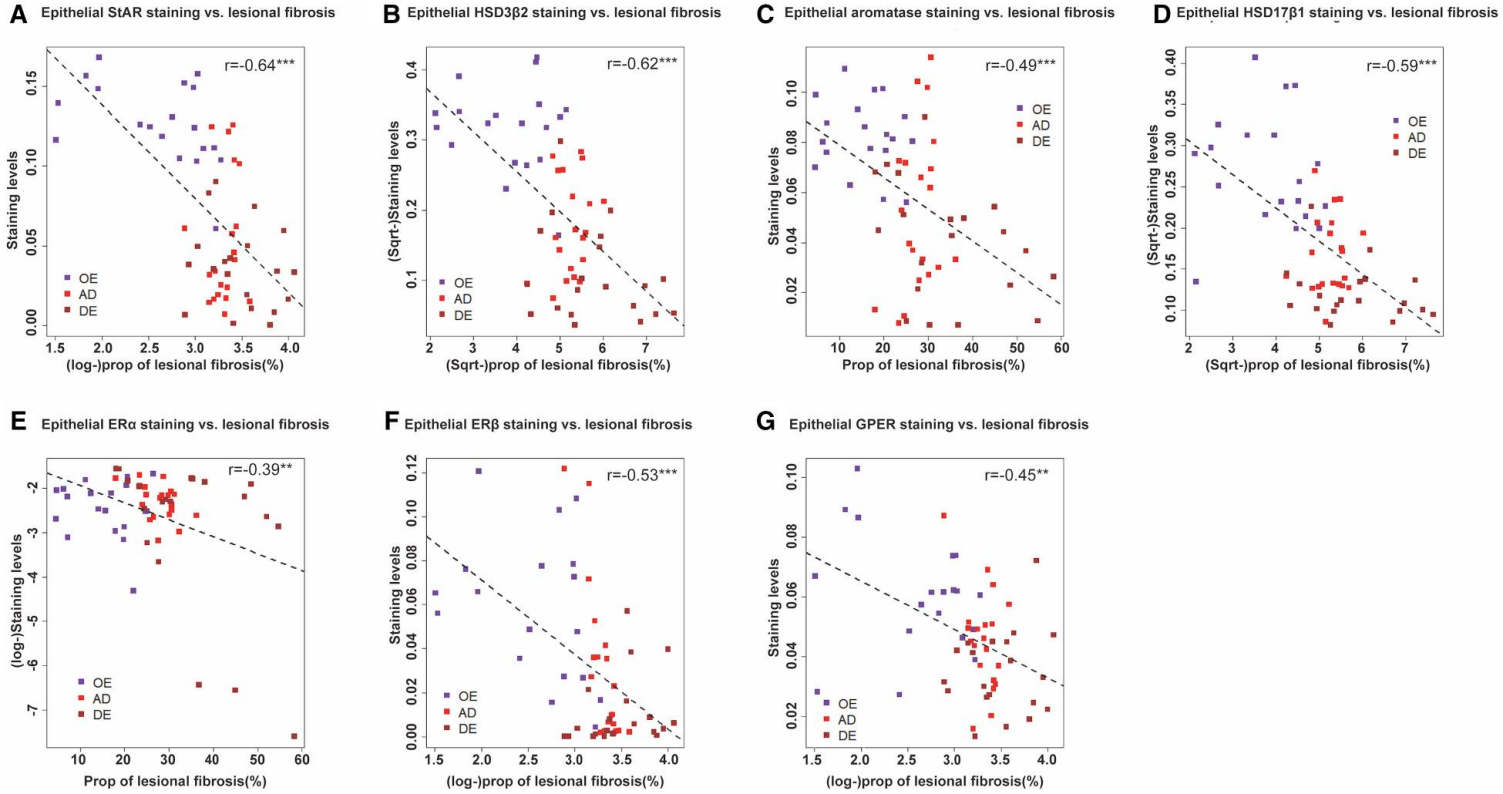
**Correlation between the extent of lesional fibrosis and epithelial staining levels of markers of estrogen signaling**. Scatter plots showing the relationship between the extent of lesional fibrosis and the staining levels of StAR (**A**), HSD3β2 (**B**), aromatase (**C**), HSD17β1 (**D**), ERα (**E**), ERβ (**F**), and G-protein coupled ER (GPER) (**G**) in ovarian endometrioma (OE), adenomyosis (AD), and deep endometriosis (DE) tissue samples. In all plots, the dashed line represents the linear regression line, and Pearson’s correlation coefficient, along with its statistical significance level, is shown. Symbols for statistical significance levels: NS: *P* > 0.05; *P < 0.05; **P < 0.01; ***P < 0.001.

**Figure 4.**
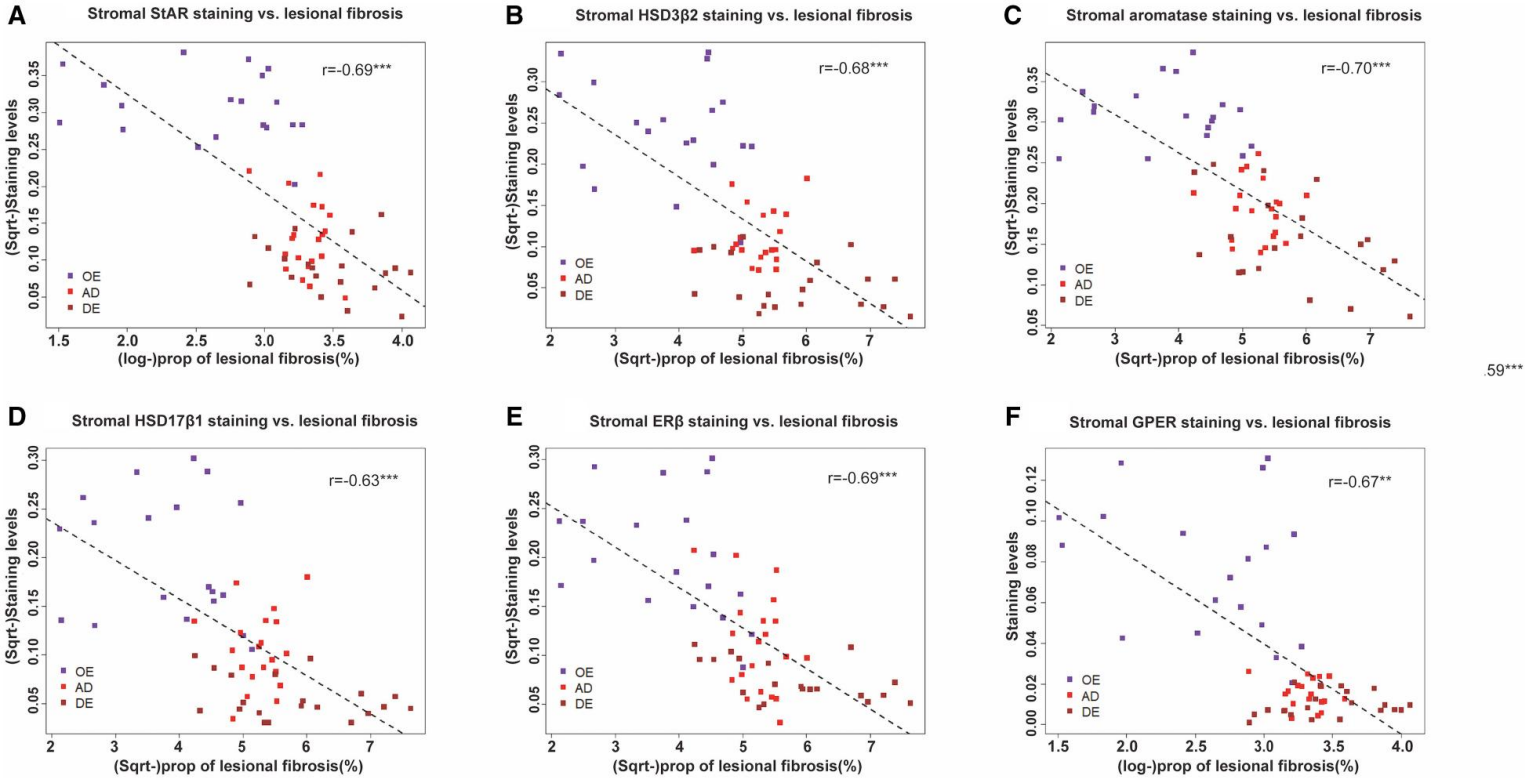
**Correlation between the extent of lesional fibrosis and stromal staining levels of markers of estrogen signaling**. Scatter plots showing the relationship between the extent of lesional fibrosis and the staining levels of StAR (**A**), HSD3β2 (**B**), aromatase (**C**), HSD17β1 (**D**), ERβ (**E**), and G-protein coupled ER (GPER) (**F**) in ovarian endometrioma (OE), adenomyosis (AD), and deep endometriosis (DE) tissue samples. In all plots, the dashed line represents the linear regression line, and Pearson’s correlation coefficient, along with its statistical significance level, is shown. Symbols for statistical significance levels: NS: *P* > 0.05; *P < 0.05; **P < 0.01; ***P < 0.001.

We found that, with the only exception of ERα in the stromal component (*r* = 0.11. *P* = 0.39), the immunostaining levels of other proteins in both epithelial and stromal components were all negatively correlated with the extent of lesional fibrosis (all *r* ≤ −0.39, *P* ≤ 0.0022; Figs 3 and 4).

Remarkably, with the only exception of ERα, the immunostaining levels of all the above proteins were mutually correlated positively in both epithelial and stromal components (all *r* ≥ 0.36, all *P*-values ≤ 0.0013). While the staining levels of ERα in the epithelial component did not correlate with other six proteins (all *r* values ranged from −0.17 to 0.11, all *P*-values ≥ 0.13), they did correlate negatively with all the other proteins in the stromal component (all *r* ≤ −0.28, all *P*-values ≤ 0.014).

More remarkably, with the only exception of ERα, the immunostaining levels of all the above proteins in both epithelial and stromal components were positively correlated with that of COX-2, mPGES-1, mPGES-2, cPGES, EP2, and EP4 (all *r* ≥ 0.23, all *P*-values ≤ 0.043). Within the epithelial component, the staining levels of StAR, HSD17β1, and ERβ, but not the others, were negatively correlated with that of 15-PGDH (all three *r*≤−0.28, all three *P*-values ≤0.012). In the stromal component, the staining levels were all negatively correlated with that of 15-PGDH except ERα (all six *r* values ≤ −0.24, all six *P*-values ≤ 0.034). In addition, the ERα staining levels in the stromal component correlated negatively with that of COX-2, m-PGES1, mPGES2, EP2, and EP4 (all *r* ≤ −0.23, all *P*-values ≤ 0.039), and positively but marginally significantly with that of 15-HPGD (*r* = 0.23, *P* = 0.056).

Thus, the ERβ and GPER staining correlated positively with those proteins critically involved in estrogen biosynthesis in both cellular components, and the estrogen signaling seemed to be correlated with the PGE_2_ signaling.

### Validation by RT-PCR and western blotting

**Figure 5.**
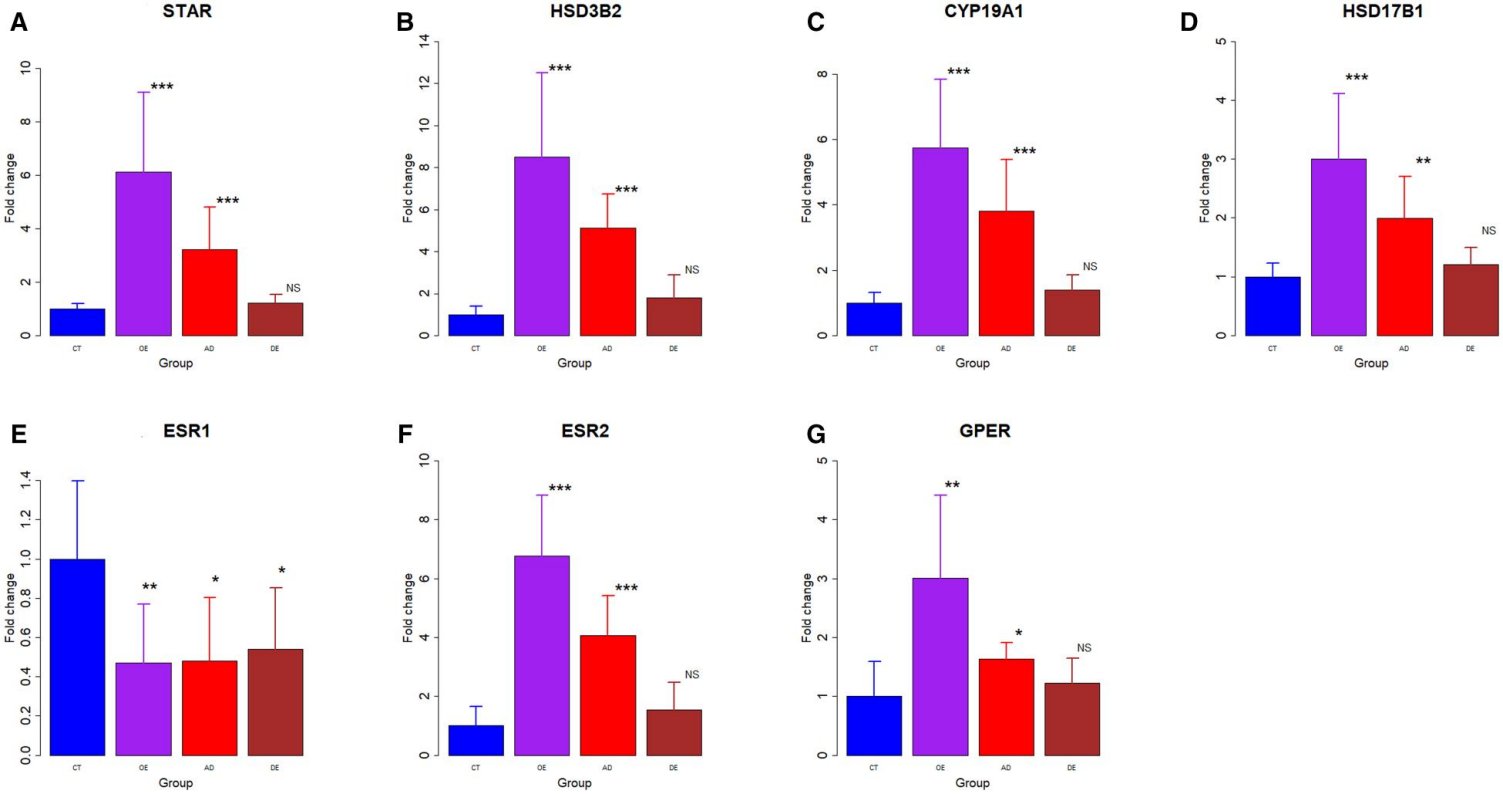
**Results of gene expression analysis. Relative fold change in gene expression of *STAR, HSD3B2, CYP19A1, HSD17B1, ESR1, ESR2*, and G-protein coupled ER (*GPER*) (A–G)**. *GAPDH* expression levels served as a loading control. All data were expressed as fold change in protein expression relative to the CT group. Symbols of statistical significance levels: NS: *P* > 0.05; *P < 0.05; **P < 0.01; ***P < 0.001 (by Wilcoxon’s rank test, compared with the CT group). Data are represented in means±SDs. CT, control endometrium; OE, ovarian endometriomas; AD, adenomyosis; DE, deep endometriosis.

To further validate our results, we evaluated both the gene and protein expression levels of genes encoding estrogen synthesis enzymes, i.e. *STAR* (encoding StAR), *HSD3B2* (encoding HSD3β2), *CYP19A1* (encoding aromatase), and *HSD17B1* (encoding HSD17β1) in the primary endometriotic stromal cells derived from OE, AD, and DE tissue samples or endometrial stromal cells derived from the control group. We found that, compared with the control group, the gene expression levels of *STAR*, *HSD17B1*, *CYP19A1*, and *HSD3B2* in both OE and AD groups were all significantly elevated (all *P*-values ≤ 0.023; Fig. 5A–D), especially OE lesions. In DE lesions, however, the expression levels of all genes were comparable to that of the control group (all *P*-values ≥ 0.083).

In addition, the gene expression levels of *ESR2* (encoding ERβ) and *GPER* were significantly elevated in both OE and AD lesions as compared with CT tissues, especially in OE lesions (all *P*-values ≤0.0498; Fig. 5F and G). In contrast, the gene expression levels in DE lesions were comparable to that of control group (both *P*-values ≥0.19; Fig. 5F and G). For *ESR1*, all three types of ectopic endometrium had significantly lower expression levels than that of the controls (all three *P*-values ≤ 0.021; Fig. 5E).

Of note, the expression levels of all four genes were mutually highly positively correlated (all *r* ≥ 0.90, all *P*-values ≤ 2.6 × 10^−12^). Furthermore, their expression levels were correlated positively with both *ESR2* and *GPER* (all *r* ≥ 0.87, all *P*-values ≤ 9.2 × 10^−11^). *ESR2* and *GPER* expression levels were positively correlated (*r* = 0.86, *P* = 2.6 × 10^−10^). Within ectopic endometrium, the gene expression levels of *COL1A1*, as reported previously (Yi *et al.*, 2025), were all highly negatively correlated with the genes critically involved in estrogen biosynthesis or coding for ERβ and GPER (all *r*’s ≤ −0.89, all *P*-values ≤ 6.2 × 10^−9^). In contrast, its expression levels were not correlated with that of *ESR1* (*r* = 0.07, *P* = 0.74).

**Figure 6.**
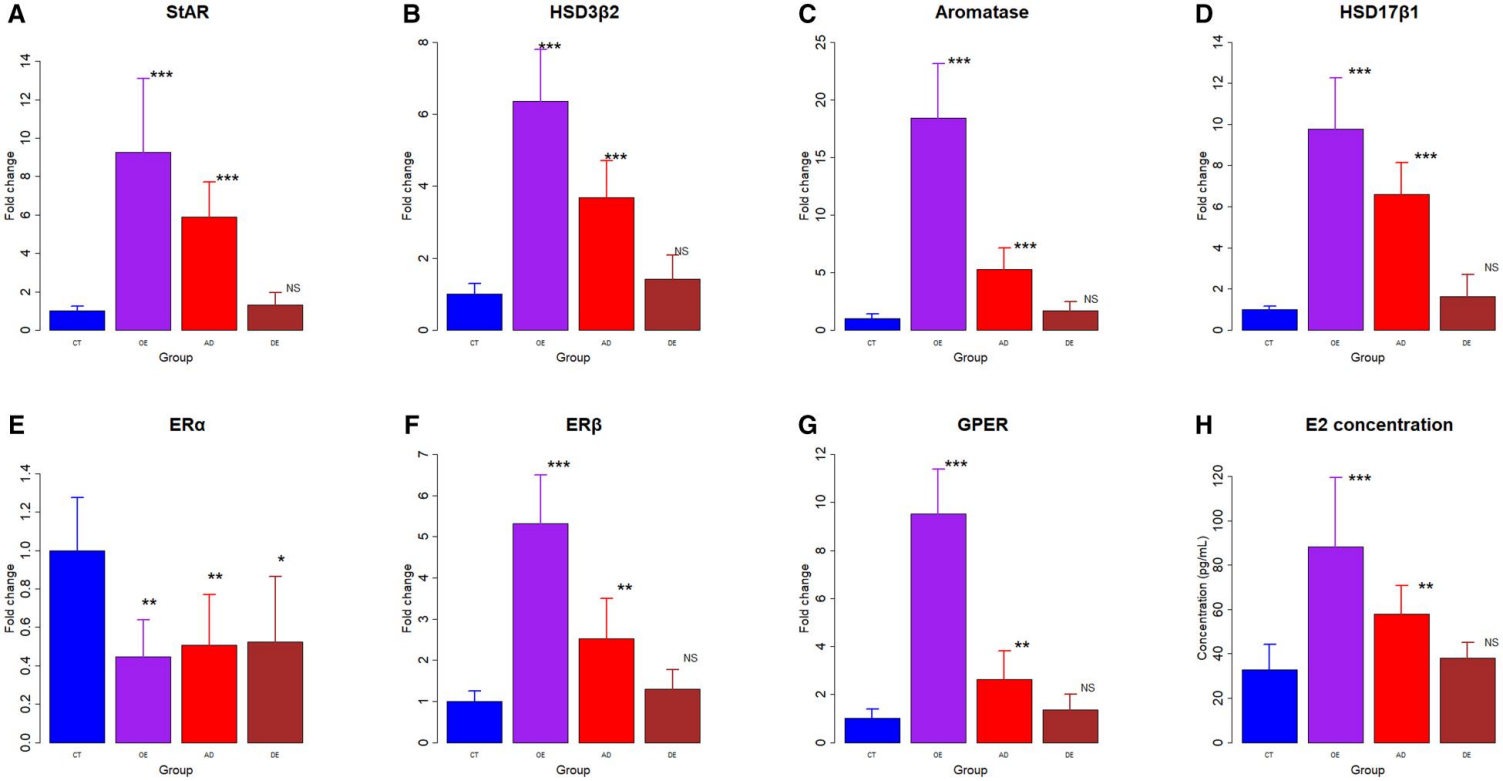
**Results of protein expression analysis and ELISA results**. Relative fold change in protein expression of StAR (**A**), HSD3β2 (**B**), aromatase (**C**), HSD17β1 (**D**), ERα (**E**), ERβ (**F**), and G-protein coupled ER (GPER) (**G**). The GAPDH expression levels were served as a loading control. All data were expressed as fold change in protein expression relative to the CT group. (**H**) Estradiol concentrations measured by ELISA in supernatant of cultured cells derived from different tissues. Symbols of statistical significance levels: NS: *P* > 0.05; *P < 0.05; **P < 0.01; ***P < 0.001 (by Wilcoxon’s rank test, compared with the CT group). Data are represented in means±SDs. CT, control endometrium; OE, ovarian endometriomas; AD, adenomyosis; DE, deep endometriosis.

We also further measured the protein expression levels of genes involved in estrogen biosynthesis as well as estrogen receptors in primary endometriotic stromal cells derived from OE, AD, and DE tissue samples and endometrial stromal cells derived from the control group. Consistent with the IHC data and the gene expression data shown above, the protein expression levels of StAR, HSD3β2, aromatase, HSD17β1, ERβ, and GPER in both OE and AD groups were all significantly elevated (all *P*-values ≤ 0.007; Fig. 6A–D, F, and G), especially OE lesions. However, the expression levels in DE lesions of all proteins were comparable to that of the control group (all *P*-values ≥ 0.065). In contrast, the ERα expression levels in all lesions were significantly lower than that of control endometrium (all three *P*-values ≤ 0.010; Fig. 6E), consistent, again, with both IHC and RT-PCR data.

Consistently, the expression levels of all four proteins involved in estrogen biosynthesis as well as ERβ and GPER were mutually highly positively correlated (all *r*  ≥ 0.87, all *P*-values ≤ 8.1 × 10^−11^). ERβ and GPER expression levels were positively correlated (*r* = 0.96, *P* < 2.2 × 10^−16^). Within ectopic endometrium, the protein expression levels of collagen I were all highly negatively correlated with the genes critically involved in estrogen biosynthesis or coding for ERβ and GPER (all *r* ≤−0.92, all *P*-values ≤ 2.3 × 10^−10^). In contrast, collagen I expression levels were not correlated with that of ERα (*r* = 0.08, *P* = 0.71).

### Estrogen concentrations in different types of ectopic endometrium

Using the same set of primary endometrial cells derived from lesions and control endometrium, we further measured the concentration of 17β-estradiol in the cell culture medium. The results were highly consistent with the expression levels of estrogen synthase. Consistent with the gene and protein expression data, the 17β-estradiol concentrations were 2.69- and 1.76-fold higher in cells derived from OE and AD lesions, respectively, as compared with the CT group (*P* = 0.00017, and *P* = 0.0011; Fig. 5H). However, in cells derived from DE lesions, the 17β-estradiol concentration was comparable to that of controls (*P* = 0.33).

Of note, the 17β-estradiol concentration correlated positively with the protein expression levels of StAR, HSD3β2, aromatase, and HSD17β1 (all *r* ≥ 0.72, all *P*-values ≤ 4.0 × 10^−6^), and with that of ERβ and GPER (both *r* ≥ 0.70, both *P*-values ≤ 7.2 × 10^−6^). However, it correlated negatively with the protein expression levels of ERα (*r* = −0.41, *P* = 0.021). Within ectopic endometrium, the protein expression levels of collagen I were highly negatively correlated with the 17β-estradiol concentration (*r* = −0.69, *P* = 0.00019; Supplementary Fig. S4).

## Discussion

In this study, we have provided multiple lines of evidence demonstrating that the estrogen signaling, in terms of both estrogen biosynthesis and the ERβ and PGER expression, is diminished concordantly with increasing lesional fibrosis. In particular, while estrogen signaling was found to be indeed activated in OE and AD lesions, it was virtually absent in DE lesions, resembling levels in control endometrium. In contrast, the ERα expression was reduced across all ectopic endometrium samples as compared with control endometrium. In addition, the elevated estrogen production that was in both OE and AD lesions contrasts with DE lesions, where its production matches that of control endometrium. The likely absence of estrogen biosynthesis in DE lesions, coupled with ERα suppression, suggests that estrogen may no longer drive lesional growth in DE lesions and lesions with high fibrotic content.

Our results of activated estrogen signaling as well as reduced ERα expression in OE and AD lesions are consistent with overwhelming data showing the same (Yamamoto *et al.*, 1993; Brandenberger *et al.*, 1999; Fujimoto *et al.*, 1999; Attar *et al.*, 2009; Mehasseb *et al.*, 2011; Heublein *et al.*, 2012; Samartzis *et al.*, 2012). Our results also align with our previous report of diminished PGE_2_ signaling in concordance with increasing fibrosis in ectopic endometrium (Yi *et al.*, 2025) since PGE_2_ signaling plays a pivotal role in the feed-forward loop in estrogen biosynthesis in endometriotic lesions (Bulun *et al.*, 2005). Higher ERβ expression in OE than AD lesions corroborates earlier reports (Liao *et al.*, 2019).

Our finding of reduced ERα expression agrees with previous reports (Brandenberger *et al.*, 1999; Mehasseb *et al.*, 2011). The ERα downregulation in endometriosis has been reported to be attributable to ERβ overexpression that suppresses ESR1 expression since the ERβ protein binds to the ESR1 gene promoter (Trukhacheva *et al.*, 2009).

Our finding of the absence of estrogen signaling in DE lesions seems to be at direct odds with the prevailing view espousing the feed-forward loop of estrogen production. However, this glaring discrepancy can be reconciled by the heavy historical reliance on OE tissue samples, likely due to the fact that OE is the most common subtype of endometriosis and, as such, the ease of procuring tissue samples. For example, the first studies reporting the overexpression of StAR, HSD3β2, aromatase, HSD17β1, ERβ, and GPER all used OE or AD tissue samples (Brandenberger *et al.*, 1999; Fujimoto *et al.*, 1999; Attar *et al.*, 2009; Mehasseb *et al.*, 2011; Samartzis *et al.*, 2012), due, likely, to less prevalent occurrence and thus the difficulty in getting DE samples. In fact, the very first study linking PGE_2_ to StAR upregulation used OE and peritoneal endometriotic tissue samples (Tsai *et al.*, 2001). Tellingly, in the very few studies that did evaluate both OE and DE lesions, higher estrogen signaling in OE lesions than DE lesions has been reported. For example, higher gene expression levels of HSD17B1, CYP19A1, and ESR2 in OE lesions than DE lesions and the comparable HSD17B1 expression in DE to normal endometrium have been reported (Huhtinen *et al.*, 2012). In fact, the same study also reported a 6-fold higher E_2_ concentration in OE lesions as compared with normal endometrium, but practically comparable E_2_ concentration in DE lesions (Huhtinen *et al.*, 2012). Similarly, the gene expression levels of CYP19A1 were reported to be the highest in OE lesions, followed by peritoneal endometriosis, and DE lesions—in that order (Heilier *et al.*, 2006).

Even though there is no local E_2_ production, the normal endometrium responds to E_2_ by activating ERα and ERβ with ERβ serving as a ‘guardian’, suppressing ERα to bridle ERα-mediated mitogenic response to E_2_ (Hapangama *et al.*, 2015). Here, we found that the local E_2_ production is elevated in OE and AD lesions, but not in DE lesions. In addition, all OE, DE, and AD lesions had reduced ERα expression, yet both OE and AD lesions, but not DE lesions, had elevated expression of ERβ as compared with control endometrium. This may be attributable to increased local E_2_ concentration that activates ERβ, which, in turn, suppresses ERα, accounting for the observed ERα downregulation but ERβ upregulation (Hapangama *et al.*, 2015). In DE lesions, however, since there is no local E_2_ production, neither ERα nor ERβ is activated.

Alternatively, due to the persistent ERβ overexpression in less fibrotic lesions, ERα would be chronically suppressed, possibly leading to ERα promoter hypermethylation (Maekawa *et al.*, 2019). In DE lesions, it is possible that they may have HDAC3 inactivation which has been reported in eutopic endometrium from women with either endometriosis (Kim *et al.*, 2019) or adenomyosis (Mao *et al.*, 2023) and may lead to ERα mRNA instability (Oie *et al.*, 2013), resulting in depressed ERα.

Our finding that the diminished or even absent estrogen signaling in DE lesions has important clinical implications. Given the current mainstay medication for treating endometriosis and adenomyosis is hormonal drugs that aim to suppress estrogen production (Barbieri, 1992), we can now understand why DE poses the biggest challenge in medical management. Aside from reduced vascularity and more epigenetic aberrations in lesions due to increased fibrosis (Liu *et al.*, 2018b), it is insensitive to external deprivation of estrogen, which also explains as why the addition of aromatase inhibitors to other hormonal drugs or, alternatively, the use of GnRH agonists does not seem to be associated with better outcomes (Vercellini *et al.*, 2018a). Also, this explains why dienogest treatment for an extended period could alleviate symptoms but has no cytoreductive effect on DE lesions (Leonardo-Pinto *et al.*, 2017). In fact, in view of the close correlation between lesional fibrosis and lesional stiffness per elastosonography (Liu *et al.*, 2018a; Ding *et al.*, 2020), our finding that the estrogen signaling diminishes concordantly with increasing lesional fibrosis calls for a re-appraisal of current treatment strategies, especially for lesions that are highly fibrotic and thus well advanced. In addition, elastography could be capitalized to guide personalized treatment strategies.

Our finding that the diminished estrogen signaling is concordant with increased lesional fibrosis also has important research implications. First and foremost, it challenges the prevailing view of universally activated estrogen signaling via the feed-forward loop in ectopic endometrium. It also casts doubts on the notion of hypomethylation of ESR2 (Xue *et al.*, 2007a) and SF-1 (Xue *et al.*, 2007b) in DE lesions, which already has been contested in a study using DE lesions (Meyer *et al.*, 2014). It remains unclear, however, what drives DE lesions if estrogen is no longer the driving force and this needs to be investigated. But this may explain a puzzling observation that lesional growth appears to be self-limited (Koninckx *et al.*, 2021). Regardless, this should inform researchers in drug development and inspire novel therapeutics for endometriosis and adenomyosis.

Second, the strength of estrogen signaling, in terms of estrogen production or ER activity, depends critically on the extent of lesional fibrosis, a proxy of the developmental stage of the lesions (Zhang *et al.*, 2016; Guo, 2018). Hence, while on average the estrogen signaling is the strongest in OE lesions, weaker in AD lesions, and absent in DE lesions, this distinction is true only in general, but we should expect to see deviation depending on fibrotic content of OE or AD lesions.

Lastly, seeing through the lens of our results, several apparent conundrums or controversies in the literature could be explained. While both endometriosis and adenomyosis are well-regarded as estrogen-dependent diseases, there are actually controversies regarding the activation status of some particular enzymes involved in estrogen biosynthesis and metabolism. For example, whether aromatase is overexpressed in endometriosis is hotly debated (Bulun, 2009; Colette and Donnez, 2009). Similarly, there are also conflicting data on ESR2 promoter hypomethylation in OE lesions (Xue *et al.*, 2007a; Meyer *et al.*, 2014; Maekawa *et al.*, 2019). In these cases, both sides could be right, but the extent of lesional fibrosis in their samples may have varied greatly yet went unaccounted for, resulting in conflicting findings.

Our study has several strengths. First, we quantitated the extent of tissue fibrosis and evaluated the genes/proteins involved in estrogen signaling against the extent of fibrosis. Since the extent of lesional fibrosis is a proxy for lesional developmental stage (Guo *et al.*, 2015; Liu *et al.*, 2016; Shen *et al.*, 2016; Zhang *et al.*, 2016), its quantification provides a yardstick with which we can compare all ectopic endometrium over their entire spectrum. Second, we evaluated three different types of ectopic endometrium along with control endometrium, which, on average, exhibit differential extent of tissue fibrosis. This provides an excellent opportunity to compare all three different disease entities using the same yardstick that is intrinsic and fundamental to all lesions, helping us to gain more insight into the molecular underpinning of endometriosis/adenomyosis. Third, we capitalized our previous results on PGE_2_ signaling and correlated our current data to the PGE_2_ data, yielding a better perspective. Lastly, we employed several methods, IHC, RT-PCR, and western blotting, to evaluate the genes/proteins in estrogen signaling, as well as ELISA to quantitate E_2_ concentration in stromal cells derived from different tissues. Remarkably, the results were quite consistent.

Our study also has several limitations. First and foremost, while we demonstrated diminished estrogen signaling concordant with increased fibrosis in lesions, we only provided strong suggestive evidence linking lesional fibrosis and estrogen signaling, and we did not provide further mechanistic data. More research in this regard is warranted. Second, while we in this study evaluated several genes/proteins known to be key to estrogen signaling, we did not evaluate some other genes/proteins that are also involved in estrogen signaling, such as other members of the HSD17B family. For example, HSD17β2 preferentially catalyzes the oxidation of E2 to estrone, a less potent estrogen, which has been shown to be depressed (Zeitoun *et al.*, 1998) but this is not without controversy (Carneiro *et al.*, 2007; Smuc *et al.*, 2007). Among other HSD17B enzymes catalyzing oxidative reactions, HSD17B6 has been shown to be negatively correlated with endometrial intra-tissue E_2_ concentration, which has been shown to be elevated in DE (Huhtinen *et al.*, 2012). Further investigation is needed to clarify these issues. Third, this study used exclusively diffuse AD samples. While this may have eased sample collection and removed possible heterogeneity between different types of AD, it may have restricted the generalizability of our findings. Future validation would be warranted in this regard, but it could be argued that, given that our finding that estrogen signaling is determined mostly by the lesional fibrotic content irrespective ectopic endometrium entities or subtypes, we believe that our results should also stand for focal AD. Lastly, the sample size for each subtype of ectopic endometrium is moderate, even though collectively the overall size is reasonably large. Traditionally, the first reports on estrogen signaling in ectopic endometrium were based on moderate sample sizes. For example, the first report of aromatase upregulation used 17 lesion samples and 7 control endometrial samples (Noble *et al.*, 1996), while that on ERα used 9 and 11 samples, respectively (Brandenberger *et al.*, 1999). The small sample sizes used in these studies reflect the challenges in procuring enough tissue samples, especially those of control samples. However, since we used different but somewhat complementary methods and still got rather consistent results, we believe that our results are genuine and credible.

To conclude, we have shown that the estrogen signaling in ectopic endometrium diminishes as lesional fibrosis increases. While in OE and AD lesions the estrogen signaling is upregulated, the signaling is comparable to normal endometrium in DE lesions. Our results shake up the prevailing view of activated *in situ* estrogen signaling in ectopic endometrium of all kinds via the feed-forward loop. As such, there is a need to re-appraise our treatment strategies, especially for lesions that are highly fibrotic and thus well advanced. Conceivably, our findings can be capitalized to help choose the best treatment modality and to inspire novel therapeutics for endometriosis and adenomyosis.

## Supplementary Material

hoaf028_Supplementary_Data

## Data Availability

The data presented in this study are available from the corresponding author upon written request specifying the purpose of obtaining the data.
