# Supplementary material for: Progressively diminished estrogen signaling concordant with increased fibrosis in ectopic endometrium
Source: Hum Reprod Open. 2025 May 15;2025(3):hoaf028. doi: 10.1093/hropen/hoaf028 (PMC12165729; doi:10.1093/hropen/hoaf028)
Supplement: hoaf028_Supplementary_Data [file hoaf028_supplementary_data.docx]

**Supplementary information**

**Progressively Diminished Estrogen Signaling Concordant with Increased Fibrosis in Ectopic Endometrium**

**Jichan Nie, Yunhua Yi, Xishi Liu, Sun-Wei Guo**

**Supplementary Table S1. List of primers used in the RT-PCR analysis.**

| **Gene** |  | **Sequence** |
| --- | --- | --- |
| GAPDH | Forward | 5’-GCACCGTCAAGGCTGAGAAC-3’ |
|  | Reverse | 5’-TGGTGAAGACGCCAGTGGA-3’ |
| STAR | Forward | 5’-TGTACCCACCTAAAACCATC-3’ |
|  | Reverse | 5’-CCCATAAAGCAAGACTTCTC-3’ |
| HSD3B2 | Forward | 5’-GCCACACAGTCACATTATCA-3’ |
|  | Reverse | 5’-ACTCCACGGTTTTCTGCTT-3’ |
| CYP19A1 | Forward | 5’-TCTCGATTCGGCAGCAAACT-3’ |
|  | Reverse | 5’-GGGCCTGACAGAGCTTTCATA-3’ |
| HSD17B1 | Forward | 5’- GTTTATTGCGCCAGCAAGTT -3’ |
|  | Reverse | 5’- CCCAACACCTTCTCCATGA -3’ |
| ESR1 | Forward | 5’-TGTGCAATGACTATGCTTCA-3’ |
|  | Reverse | 5’-GCTCTTCCTCCTGTTTTTA-3’ |
| ESR2 | Forward | 5’-GCTTCGTGGAGCTCAGCCTG-3’ |
|  | Reverse | 5’-AGGATCATGGCCTTGACACAGA-3’ |
| GPR30 | Forward | 5’-ACACACCTGGGTGGACACAA-3’ |
|  | Reverse | 5’-GGAGCCAGAAGCCACATCTG-3’ |

**
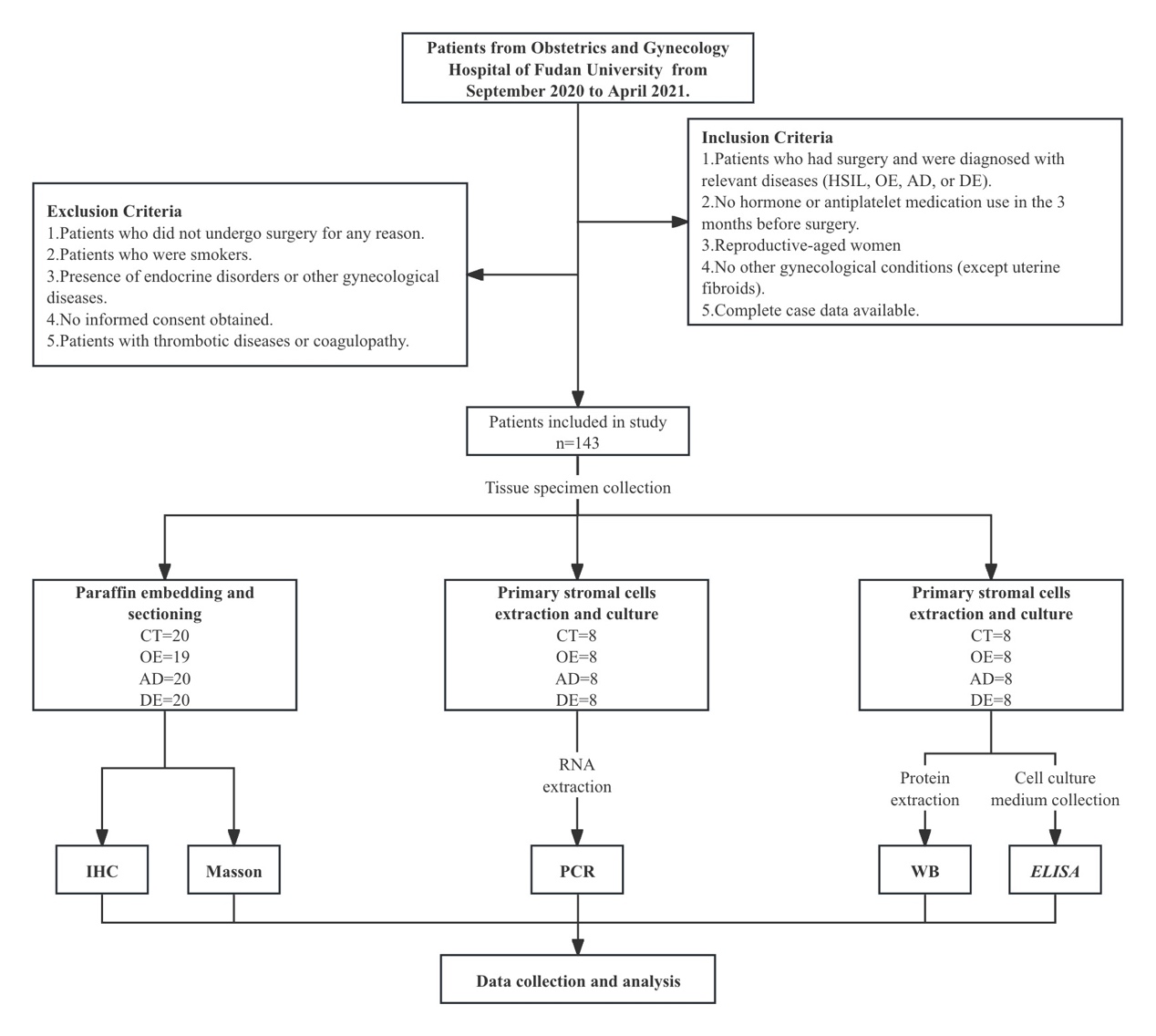
**

**Supplementary Figure S1.** Flow chart illustrating the study design, along with the inclusion and exclusion criteria. We included a total of 123 patients who underwent surgery and were pathologically diagnosed with HSIL, OE, AD, and DE at the Obstetrics and Gynecology Hospital of Fudan University between September, 2020 and April, 2021. Ectopic endometrial tissues or eutopic endometrial tissue samples were collected for the study.

**
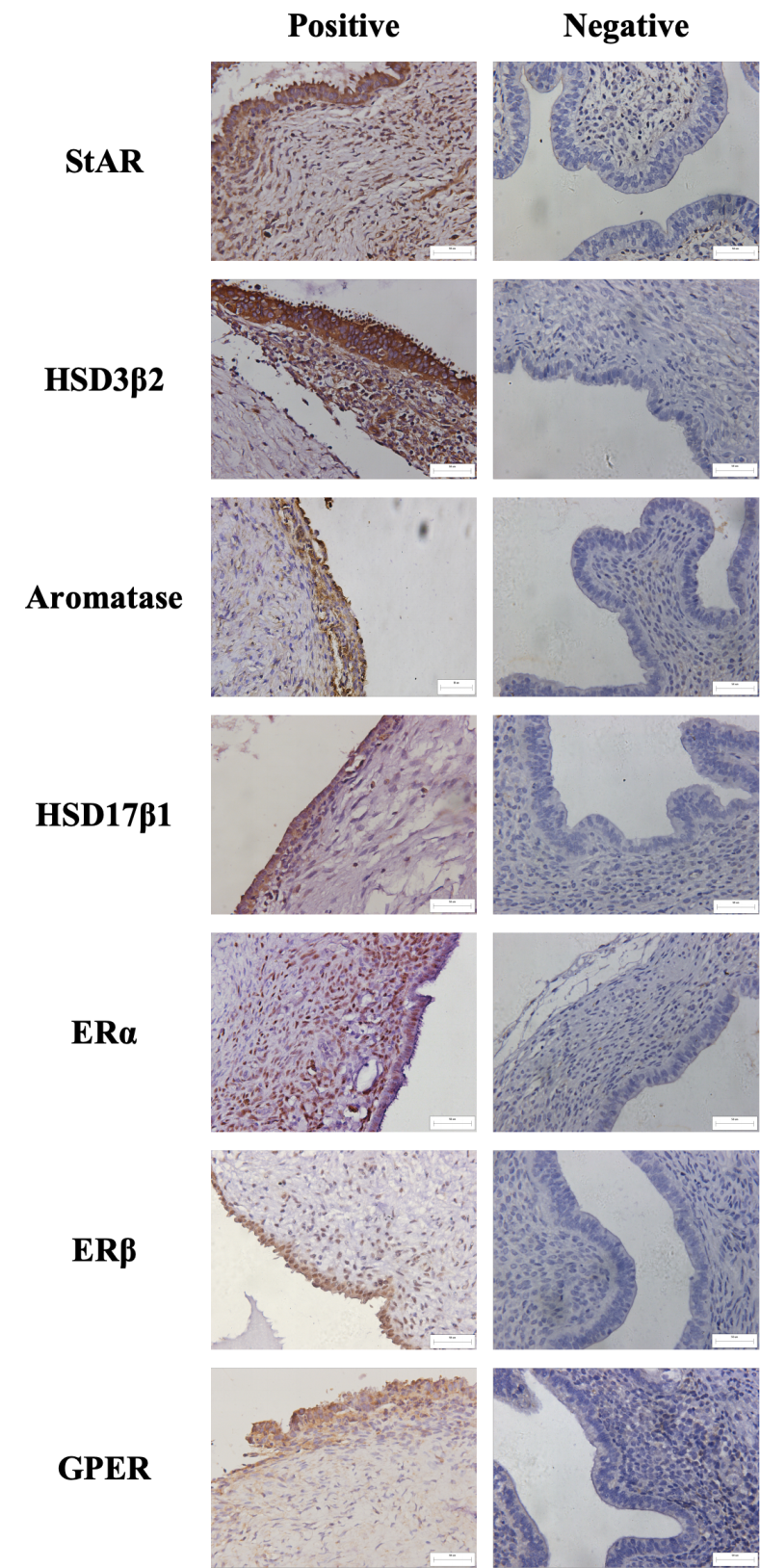
**

**Supplementary Figure S2.** Positive and negative controls for immunohistochemistry. As positive controls, we used ectopic endometrium from ovarian endometriosis as positive controls for StAR, HSD17β1, Aromatase, HSD3β2, ERα, ERβ, and GPER.


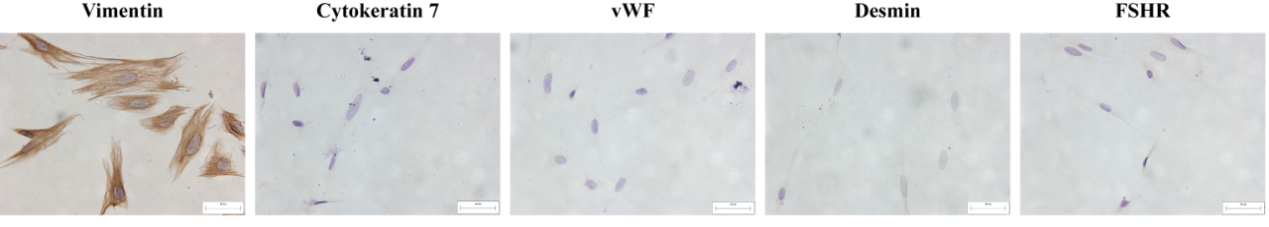


**Supplementary Figure S3. The representative photomicrographs of the immunostaining of primary stromal cells.** Purity identification of primary mesenchymal cells at passage 3: Vimentin (+): used to confirm mesenchymal cells; Cytokeratin 7 (-): used to exclude epithelial cells; vWF (-): used to exclude endothelial cells; Desmin (-): used to exclude muscle cells; FSHR (-): used to exclude ovarian granulosa cells.


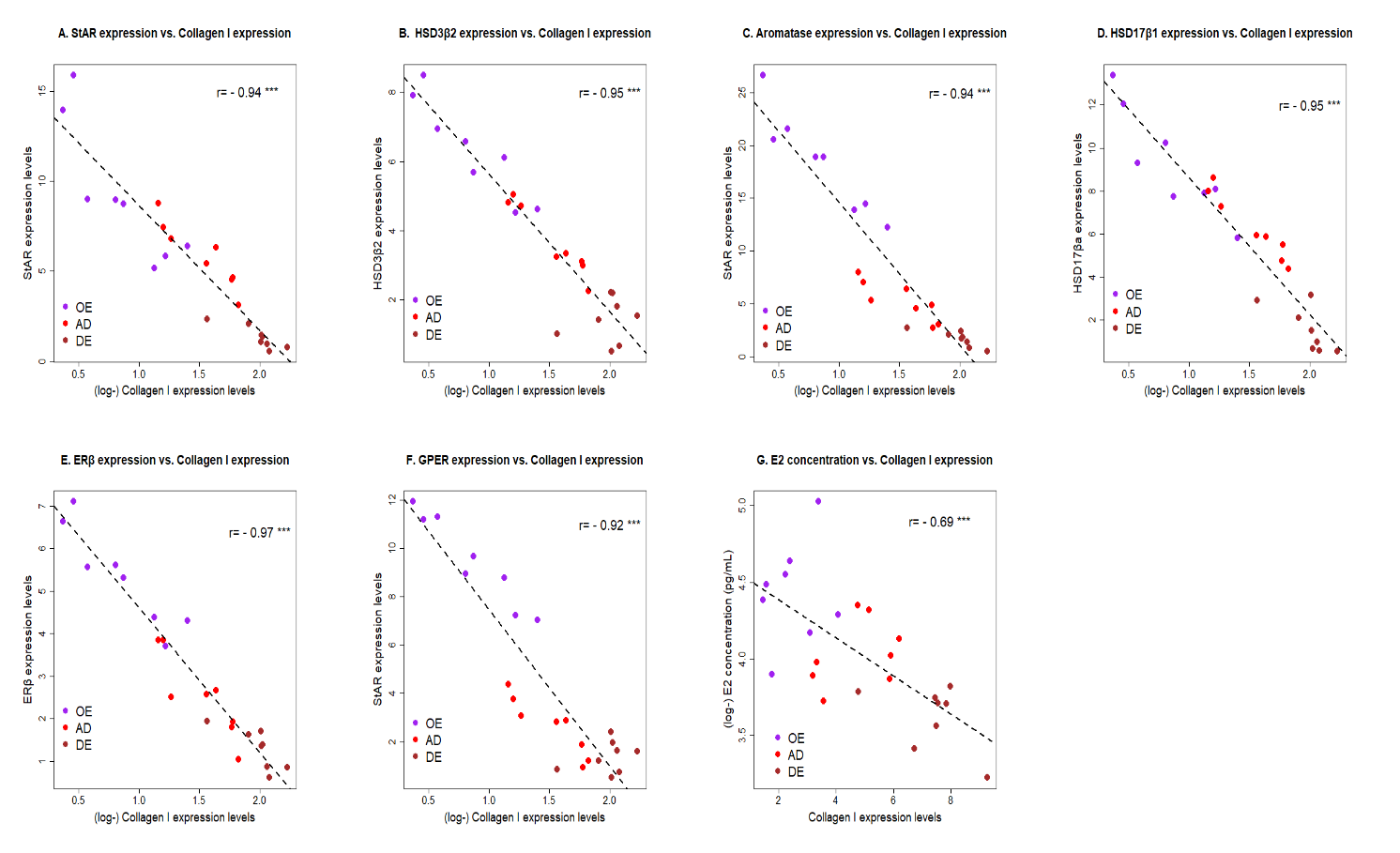


**Supplementary Figure S4. Scatter plots showing the negative correlation between protein expression levels of collagen I and those involved in estrogen signaling.** (A) StAR and collagen I. (B)HSD3β2 and collagen I. (C) Aromatase and collagen I. (D) HSD17β1 and collagen I. (E) ERβ and collagen I. (F) GPER and collagen I. (G) 17β-estradiol (E_2_) concentration and collagen I. In all plots, the dashed line represents the linear regression line, and Pearson’s correlation coefficient, along with its statistical significance level, are shown. Symbol for statistical significance levels: ***: p < 0.001.
